# Community pharmacists' views on providing a reproductive health service to women receiving opioid substitution treatment: A qualitative study using the TDF and COM-B

**DOI:** 10.1016/j.rcsop.2021.100071

**Published:** 2021-09-21

**Authors:** N. Alhusein, J. Scott, J. Neale, A. Chater, H. Family

**Affiliations:** a Population Health Sciences, University of Bristol, Bristol, UK; b Department of Pharmacy & Pharmacology, University of Bath, Bath, UK; c National Addiction Centre, King's College London, London, UK.; d Centre for Social Research in Health, University of New South Wales, Sydney, Australia; e Centre for Health, Wellbeing and Behaviour Change, University of Bedfordshire, Bedford, UK; f UCL School of Pharmacy, Centre for Behavioural Medicine, BMA House, Tavistock Square, London WC1H 9JP, UK

**Keywords:** Community pharmacists, Reproductive health, Opioid substitution treatment, Theoretical domains framework, Capability-opportunity-motivation to behaviour (COM-B) model, Health services for women, OST, Opioid Substitution Therapy, SC, Supervised Consumption service, RH, Reproductive Health, CPs, Community pharmacists, LARC, Long-Acting Reversible Contraceptives, EHC, Emergency Hormonal Contraception, BCW, Behaviour Change Wheel, COM-B, Capability-Opportunity-Motivation to Behaviour model, TDF, Theoretical Domains Framework

## Abstract

**Background:**

The absence of menstruation is common in women who use drugs. This can give a belief that conception is unlikely. When stabilised on Opioid Substitution Treatment (OST), fertility often returns, initially without realisation as ovulation precedes menstruation. This leaves women vulnerable to unplanned pregnancies. Community pharmacists (CPs) are frequently in contact with this patient group through the Supervised Consumption of OST service. This provides a timely opportunity to provide reproductive health (RH) advice. The aim of this study was to investigate pharmacists' views on providing a RH service to women receiving OST.

**Methods:**

Twenty semi-structured interviews based on the Capability-Opportunity-Motivation to Behaviour (COM-B) model and the Theoretical Domains Framework (TDF) were conducted between 2016 and 2017. Data analysis involved deductive coding using the TDF domains. The TDF domains were mapped onto the elements of the COM-B and used in the second step to create the framework and chart the data. The third step involved re-reading and clustering the codes, and inductive themes were generated to explain the data in depth.

**Results:**

Nine of the 14 TDF domains, mapped into five elements of the COM-B, were identified. Five inductive themes were generated: 1) The pharmacists' experience and knowledge of reproductive health (RH) needs of women receiving OST, 2) The pharmacists' approach to providing advice, 3) The pharmacists' perception of the relationship with women receiving OST, 4) Social influences, and 5) Environmental factors. Community pharmacists feared causing offense to women receiving OST and described requiring cues as to when the service was needed. Pharmacists' highlighted a power imbalance in the relationship with women receiving OST. This could influence how receptive this patient group would be to pharmacy RH interventions.

**Conclusions:**

CPs' concerns of providing RH service could hinder a proactive service provision. Supporting good rapport and providing a structured consultation would increase the accessibility of such a service.

## Highlights

• Reproductive health advice is triggered by social or physical cues from women.
• Provision of this advice is subject to the reflective thinking of the provider.
• A power imbalance characterizes supervised opioid substitution treatment.
• Good rapport and a structured consultation were seen to increase the accessibility.

## 1 Introduction

In the UK, community pharmacists often supervise the consumption of Opioid Substitution Therapy (OST) (e.g. methadone, buprenorphine) in their pharmacies, especially in the early stage of opioid use disorder treatment.,^1^^,^^2^ This supervised consumption service (SC)^3^ provides the service user with regular contact with a health care professional (the pharmacist), more frequently than others involved in their care. In this context, the Department of Health described that role as *“providing an opportunity for the pharmacist to build a therapeutic relationship with the patient that is beneficial to promote health and harm reduction,* (p.102)”.^4^ However, little research has been conducted to illustrate how pharmacists contribute to the general wellbeing of SC patients.^5^

For women receiving OST, their reproductive health (RH) is significantly compromised compared to those from the general public.6., 7, 8., 9 For example, 35% of women who receive OST use contraception compared to 75% of those in the general population.6., 7, 8. Additionally, a retrospective cohort study found that women receiving OST have an age-matched termination rate of 0.46 per woman vs 0.025 for the general population.^7^ Poor diet, weight loss, amenorrhea and anovulation are common in women who are addicted to opioids.^9^ The absence of menstruation can lead women to believe they are unable to conceive.^9^ However, when stabilised on OST, their fertility often returns without their realisation because ovulation precedes menstruation.^6^^,^^10^ This leaves women receiving OST vulnerable to unplanned pregnancies which might have significant consequences on the mother and child. The Department of Health recommends that community drug teams and OST prescribers should provide contraception advice.^11^ However, this advice is not routinely given as fewer than 40% of women attending community drug teams in London reported discussing contraception with their drug worker or general practitioner.^6^

Public Health England strongly recommends the expansion of the CPs' role in delivering sexual and reproductive health in general,^4^ as CPs are accessible to all including people living in deprived communities who often face health inequalities. The recommendations include supplying oral contraception as well as ensuring referral for a timely provision of long-acting reversible contraceptives (LARCs). Many pharmacies are already engaging in providing services related to RH to the general public including emergency hormonal contraception (EHC), Chlamydia screening and treatment, pregnancy testing, condom distribution and sexual health promotion.^12^

The purpose of this study was to explore the acceptability and feasibility of the potential role expansion for CPs with a view to informing the design of a future intervention to provide women receiving OST with reproductive health (RH) service.

## 2 Methods

A qualitative study using semi-structured interviews was undertaken. The execution of this manuscript was conducted to comply with the COREQ (Consolidated criteria for reporting qualitative research) guidelines for reporting qualitative research.^13^

### 2.1 Participant recruitment

Twenty community pharmacists (CPs) were recruited from pharmacies across London and parts of the Southwest of England between September 2016 and May 2017.

The sample size was chosen to provide a medium sized sample for Framework Analysis and one which was practical within the timeframe for this study.^14^^,^^15^ Recruitment was opportunistic due to challenges in recruiting CPs into research studies due to workload and difficulties in finding a locum cover. CPs were therefore offered a two-hour locum fee (£70) to cover their practice time while participating in the study and as an acknowledgement of participation.^16^^,^^17^ Their travel expenses were also paid if they travelled to meet the researcher.

### 2.2 Materials

**Fig. 1:**
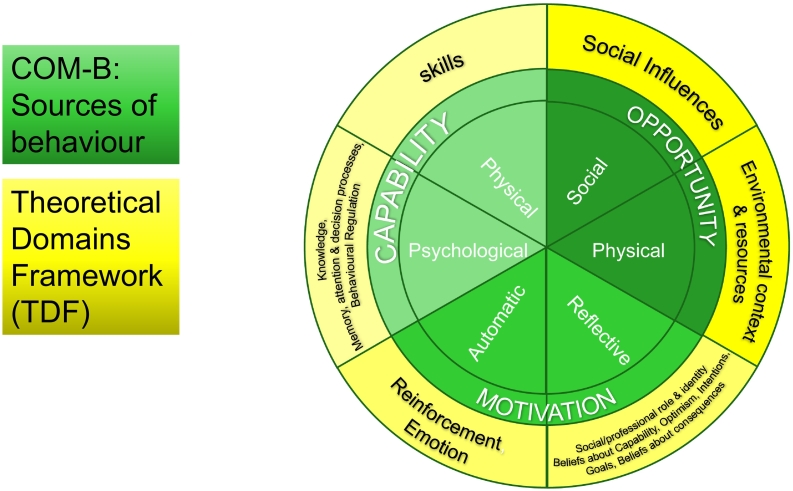
A flow chart illustrates the COM-B system and the TDF domains, based on References 19 and 34.^19^^,^^34^

The topic guide development (and later the analysis) was informed by the (COM-B) model,^18^ and the TDF (see Fig. 1),^19^^,^^20^ in consultation with a Project Advisory Group (included clinicians and patient representatives). Growing evidence supports the use of theory when understanding a behaviour. The COM-B model was designed to facilitate a behavioural diagnosis by understanding six determinants of behaviour (psychological capability, physical capability, automatic motivation, reflective motivation, social opportunity, and physical opportunity).^18^ This then is linked with intervention strategies for behaviour change.^18^^,^^21^ The TDF is a framework originally synthesised from 33 theories of behaviour and behaviour change which draws together cognitive, affective (emotion), environmental and social influences on behaviour.^19^^,^^20^ The TDF was later mapped into the six elements of the COM-B model as to give a more detailed insight to the target behaviour (see Fig. 1).^18^^,^^20^^,^^21^ The target behaviour in our study was the CPs' provision of RH service to women receiving OST. This approach to intervention development design is widely used,^22^ and has been used in previous research on contraception interventions.^23^^,^^24^ The topic guide covered CPs' experience of the SC service and the RH needs of women receiving OST. It also covered CPs' views on the design of a future RH service (Supplementary file 1) The topic guide was piloted once and since no issues were reported from the interviewee, the piloted interview was incorporated to the study. In this paper, we report the first three topics covered in the topic guide. Service design and training needs are reported separately in-conjunction with data from women receiving OST as these describe the next step of the intervention development.

### 2.3 Procedure for data collection

CPs received a study information pack through the post prior to the interview date. At the start of the interview, CPs completed and returned their written consent form. Interviews took place either by phone (*n* = 5) or face-to-face (*n* = 15) at a time and place of choice of the participant. No repeated interviews took place and transcribed interviews were not offered to interviewees to check due to not having the time capacity to do so. Interviews were conducted and audio recorded by (NA), a post-doctoral female pharmacist researcher, PhD, (17 interviews) and (HF), a female lecturer, chartered psychologist and health psychology researcher, PhD, (three interviews). Both interviewers are trained qualitative interviewers. The research team comprised researchers with backgrounds in health psychology applied to pharmacy (HF, AC), addiction science (JN, JS, NA), and pharmacy (JS, NA).

### 2.4 Ethics

The study was reviewed and approved by the Greater Manchester West Research Ethics Committee (REC Reference 16/NW/0376). Health Research Authority approvals to conduct the research were given (IRAS Ref: 20049).

### 2.5 Analysis

The recorded semi-structured interviews were anonymously transcribed verbatim, then analysed using NVivo qualitative data analysis software (Version 11 (mac), QSR International, Melbourne, Australia). Data were analysed using a combination of framework analysis,^25^^,^^26^ and thematic analysis.^27^ This enabled the identified themes, issues, or discussion points to be explored in depth via thematic analysis while using the COM-B model as an explanatory framework and to help build a future intervention.^28^, ^29^, ^30^, ^31^, ^32^, ^33^ The first step in the analysis involved NA reviewing the transcripts through the stages of familiarization and deductively coding the transcripts using the 14 TDF domains. Inductive codes were also added to capture key elements of the pharmacists' interviews which were not adequately described by the TDF domains (e.g. CPs' past experience of RH needs of women receiving OST, CPs' perception of the characteristics of women receiving OST). NA coded all the transcripts. At the start of coding NA and HF coded two transcripts, codes were compared, and discrepancies discussed to ensure that coding to the TDF was consistent. The next step of the framework analysis involved charting the data and mapping the TDF domains to the COM-B model. The third step of analysis drew from the thematic analysis approach of Braun and Clarke,^27^ with the purpose of developing inductive themes that explained in depth pharmacists' views on providing RH service to women receiving OST. This step was achieved by re-reading, reviewing and clustering the identified TDF codes, the COM-B constructs and the identified inductive codes to form inductive themes and sub-themes, and then naming the themes.

## 3 Results

### 3.1 Demographics

Twenty community pharmacists (CPs) took part in the study, 10 from London (*n* = 2 female, *n* = 8 male), and 10 from the southwest of England (*n* = 4 female, *n* = 6 male). The average age of the CPs was 39 ± 9 years old (missing data for 2 CPs). Over half of the interviewees (*n* = 12) worked in independent pharmacies and there was one locum. Years of practice ranged between 2.5 and 35 years and CPs originated from eight different ethnicities. We asked the CPs how many female OST clients they currently had, and it ranged between 1 and 12, with some CPs declining to answer. Table 1 reports the participants' characteristics in detail. The average time of the interviews was 70 ± 19 min.

**Table 1:** Participant characteristics.

ID	Age	Gender	Ethnicity	Type of pharmacy	Years of practice	Number of women, attending the pharmacy
Pharm 1	50	Female	White British	Large multiple	28	1 (and one recovered)
Pharm 2	nd⁎	Male	North African	Large multiple	7	Between 50 and 60 clients, females' number is unknown
Pharm 3	nd⁎	Male	White British	Large multiple	27	~12
Pharm 4	35	Male	Black African	Independent	12	1 (and 2 recovered)
Pharm 5	39	Male	Any other white background	Locum	11	Depends on the pharmacy
Pharm 6	51	Male	White British	Large multiple	12	7
Pharm 7	36	Female	Any other white background	Independent	12	~10
Pharm 8	36	Male	Any other white background	Independent	12	Large number, preferred not to say
Pharm 9	38	Female	White British	Large chain	12	~6
Pharm 10	31	Female	Chinese	Independent	7	4
Pharm 11	32	Female	North African	Large multiple	3	1
Pharm 12	35	Male	Indian	Independent	5	1
Pharm 13	33	Male	Black British-African	Independent healthy living pharmacy	8	3
Pharm 14	53	Male	Black British-African	Independent	23	3
Pharm 15	59	Male	Black African	Independent	35	5
Pharm 16	42	Male	Black African	Independent	10	6
Pharm 17	33	Female	North African	Independent	6	6
Pharm 18	27	Male	Black British-African	Large multiple	2.5	7
Pharm 19	32	Male	Indian	Independent healthy living pharmacy	4	6
Pharm 20	45	Male	British Asian	Independent	23	2

⁎ nd: no data.

### 3.2 TDF and COM-B behavioural diagnosis – determinants of the CPs' provision of RH service to women receiving OST

**Fig. 2:**
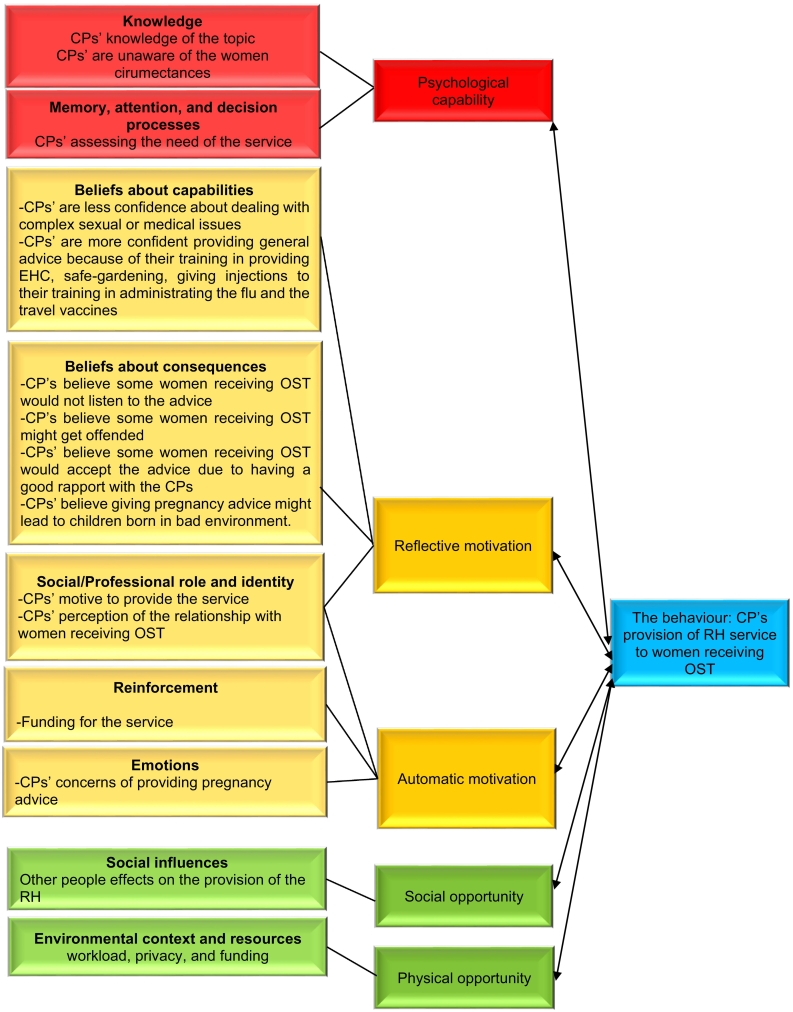
Combined COM-B and TDF analysis of the determinants of the CPs' provision of RH service to women receiving OST.

Framework analysis led to the identification of nine of the 14 TDF domains: knowledge; memory, attention and decision making; beliefs about capabilities; beliefs about consequences; social /professional role and identity; emotion; reinforcement, social influences; environmental context and resources. These domains were then mapped onto five elements of the COM-B model: psychological capability; reflective motivation; automatic motivation; social opportunity; and physical opportunity. Fig. 2 shows the mapping process with the identified TDF domains and COM-B constructs presented.

### 3.3 Inductive themes

**Fig. 3:**
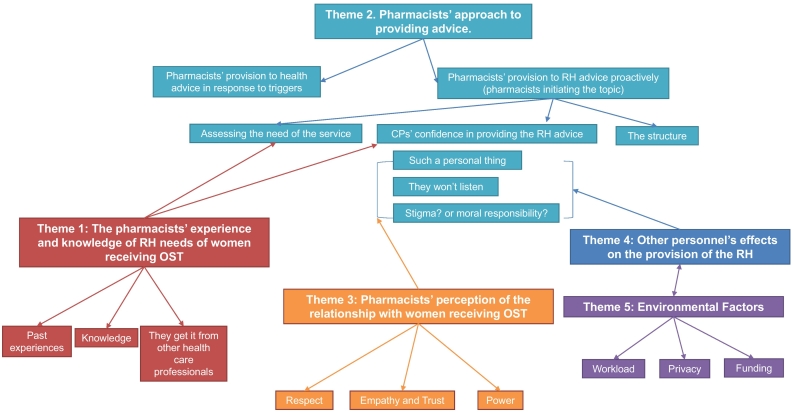
Thematic map presenting the five inductive themes generated from the data. Each colour represents a theme and subthemes within that theme. The arrows represent how these themes influence each other. The subthemes highlighted in green show the thought process of the CPs around providing RH advice. It starts with the CPs responding to triggers (women asking), assessing the need for the service, then reflecting on their confidence and outcome expectancy of such service, balancing between their own prejudices and their professional duties, and finally concluding that a structure is needed to provide the service. (For interpretation of the references to colour in this figure legend, the reader is referred to the web version of this article.)

Five themes were inductively generated: 1) The pharmacists' experience and knowledge of reproductive health (RH) needs of women receiving OST, 2) The Pharmacists' approach to providing advice, 3) The pharmacists' perception of the relationship with women receiving OST, 4) Social influences, and 5) Environmental factors (see thematic map in Fig. 3).

These themes are presented below with the related COM-B constructs and TDF domains underneath (shown in brackets (COM-B; [TDF])). Anonymised quotations from CPs are presented, with their participant number.

## 4 Theme 1. The pharmacists' experience and knowledge of reproductive health (RH) needs of women receiving OST

*(COM-B: Psychological Capability and Reflective Motivation;* [*TDF domains: knowledge; Beliefs about consequence*])

Many CPs had experience of providing OST for women who were pregnant (planned and unplanned) or who needed support with their reproductive health (RH) needs.

*“She said “I seem to have put on weight but I am not eating more... she said that she hadn*'*t had a period and then I asked whether she had any sexual... she can*'*t remember... I offered her a pregnancy test... it came back positive”* CP. 2

*“She was very happy and she was looking forward to having her baby and by the time [of birth], she was off… she was not on methadone”* CP. 12

Most CPs reported that they did not have enough knowledge about OST effects on fertility [TDF: Knowledge]. Their advice usually would include to take folic acid, maintain a healthy diet, stop smoking, avoid alcohol, and being checked for sexually transmitted diseases (STD). Most CPs felt they would be able to talk about the different contraception methods but felt unable to advise on the most suitable method for any given individual [TDF: knowledge]. Some said they would recommend the use of long-acting reversible contraception (LARC; e.g. intrauterine device, contraceptive implant, contraception injection) for this patient group, because women receiving OST were perceived as likely to forget to take oral contraception (‘the pill’) regularly [TDF: Beliefs about consequences].

*“I know that probably oral contraception might not be umm a very efficient method for her because she might forget to take… But err I think the one of the implants or the intrauterine devices because they will be better suited for her” CP. 2*

Most CPs did not know whether women used contraception [TDF: Knowledge] and they speculated that women must have had RH advice from other health care professionals.

*“I don't know whether they might have got implant or anything or getting prescriptions for contraception I wouldn't know or if they go to family planning clinics”* CP. 13

## 5 Theme 2. The pharmacists' approach to providing advice

*(COM-B: Psychological Capability, Reflective Motivation, Automatic Motivation, Social Opportunity, Physical Opportunity; [TDF domains: knowledge, memory, attention and decision making; Beliefs about capability; Beliefs about consequence; emotions; social/professional role and identity; social influences; environmental context and resources])*

Most CPs indicated that they struggled with the concept of proactively providing RH advice to every woman receiving OST. Some felt that a woman should initiate the topic, or they needed an opportunity onto which they could add the topic onto. The sub-themes below map out the thought process that CPs discussed during the interview.

### 5.1 Pharmacists' provision to health advice in response to triggers

Many CPs described their approach to provide health advice generally as a response to triggers i.e. social triggers (the patient initiating the subject) [TDF: social influences] or physical triggers (an issue related to a prescription) [TDF: environmental context and resources]. This response (COM-B: automatic motivation) was motivated by the CPs' sense of their professional role and identity as health care providers [TDF: social/professional role and identity].

*“It's not something that's always in my mind to be honest… when I talk to patients it's probably when I see their medication history if they're taking something that would interact, but if I'm not aware that medication interact with their pill, I wouldn't approach the subject unless they ask about it”* CP. 11.

*“I mean I'll always give them advice if they ask for it, but if they expect me to just bring it out doesn't make any sense you need a trigger to initiate something”* CP. 19.

### 5.2 Pharmacists' provision to RH advice proactively (pharmacists initiating the topic)

When it was suggested that RH advice could be provided proactively (without being asked for by patients), CPs reflected on different issues:

### 5.3 Assessing the need of the service

Some CPs articulated that the woman's need for this advice should be assessed on an individual basis [TDF: memory, attention and decision processes]. However, CPs also described assessing women's need for RH advice as challenging because they often did not know the women's circumstances e.g. whether they had a partner(s), were hoping to be pregnant now or in the future, if they had contraception already or if they needed it [TDF: Knowledge]. Several factors were seen as potential indicators for the need for RH advice, e.g. age, involved in sex work, appearance, homelessness, partners [TDF: Social influences].

*“it would depend on need; we cannot give out this advice to everybody”* CP. 15.

*“Researcher: when would you give this advice to women? CP: When I thought it is needed. Researcher: when do you think it is needed? CP: So if I see if they are sexually active if, this is regarding the methadone and the substance misuse patients yeah if I sense if I see them with different people all the time or they have a regular partner still tell them”* CP. 18.

### 5.4 CPs' confidence in providing the RH advice

CPs said their experience of training for and providing other similar services (e.g. EHC, the flu vaccine) rendered them open to adopt this service (with the right training) However, several CPs said they would feel less competent in providing the advice to women receiving OST with complicated medical cases, history of sexual abuse or struggling with mental health [TDF: Beliefs about capabilities].

*“If I felt that this conversation that I couldn't have a logical conversation with the patient the patient was maybe one that had really badly managed psychotic, err you know psychological conditions I don't want to feed into something that's going to create more problems for the patient. So I think it would be the same reasons why I would not do that”* CP. 4.

*“Such a personal thing” woman receiving OST might get offended.*

CPs felt RH was a personal topic and they feared women receiving OST might be offended or uncomfortable. This was even more of a concern for male pharmacists or locums (locums were aware that SC patients were less comfortable with new faces). CPs worried that discussing RH may raise difficult emotions e.g. difficulties in getting pregnant, sexual abuse, and previous children taken into care. CPs sometimes described women as being ‘angry’, ‘intimidating’, ‘closed down’ or ‘ashamed’ and explained how this made conversations difficult. Conversely, other female OST patients were perceived as easier to talk to because they were ‘chatty’ or friendly [TDF: Emotions, Beliefs about consequences].

*“You know we have to be careful that we're not doing the one thing that the patient doesn't want… [gives examples of what women might say if CPs raised RH topic] “Are you having this conversation with me just because I take methadone””* CP. 4.

*“I think mine would be alright actually we have good chats anyway”* CP. 9.

*“They won't listen” Women receiving OST would not listen.*

Some CPs questioned whether the woman would accept the advice. CPs used expressions like *“if they listen”* or *“if they understand”*, *“not going to be receptive”* or *“if they're willing to discuss”* to describe some the perceived possible reactions. Some CPs also highlighted a belief that women tend to gain their health information from unreliable sources and not listen to health care professionals [TDF: Beliefs about consequences].

*“Some of them are just not very compliant wouldn't listen and so that's another thing that would make me reluctant, so it goes on the character of the patient”* CP. 17.

### 5.5 Stigma? or moral responsibility?

CPs expressed worry and concerns [TDF: emotions] about the consequences of the advice they might give. Some CPs were of the opinion that for women who engaged in risky behaviours, pregnancy planning advice was not appropriate as their risk behaviours could have adverse effects on the developing child [TDF: Beliefs about consequences]. As a result, CPs felt more at ease providing contraception advice.

*“From a feeling point of view, umm, I suppose it is being much more aware of their likelihood of risky behaviours and worrying what might or might not go wrong as a result of your advice... that's in a way it is always in your subconscious... Again, it is about helping them to make the right decisions for themselves and if they decide, whether it because of their own acknowledgement of their own behaviours, that it is not right to have a baby or if they are wanting support to not have a baby, then it is making sure they are getting the right support in the right way I suppose”* CP. 6.

### 5.6 The structure

Many CPs suggested that the topic should be approached through a structured consultation on wider lifestyle advice or medicines review, so as to leave the choice to the women to ask for further advice or not.

*“So, in order for me to bring such a topic umm it needs to have umm kind of properly structured consultation… you are taking a full history … and then patients see it as normal”* CP. 2.

## 6 Theme 3: the pharmacists' perception of the relationship with women receiving OST

*(COM-B: Automatic Motivation, Social Opportunity; [TDF domains: social/professional role and identity, emotions, social influences])*

Most CPs highlighted that having a good relationship with women receiving OST was central to the patient accepting the pharmacist's intervention.

*“You have to have a close relationship to tell them, one girl that she was coming a lot for the [emergency] oral contraception so I told her to go on the injection and she is on the [contraception injection brand name] at the moment”* CP. 16.

The dynamics of this relationship seemed to be influenced by several factors including the nature of the SC service (i.e., controlled, pharmacist has the power to approve/not approve the administration), the patient group characteristics (easy going vs difficult client) [TDF: social influences], and the pharmacists' perception of their professional role (involved and empathetic, vs having boundaries) [TDF: social/professional role and identity]. The interaction between these factors led to three characteristics of this relationship including a relationship built on empathy and trust, a relationship built on respect or that built on power.

### 6.1 Empathy and trust

Due to the regular contact between CPs and women receiving OST (daily to weekly), CPs described developing good rapport with women [TDF: emotions]. CPs talked about providing physical health care, social support and keeping an eye on their wellbeing (e.g. reporting concerns back to prescribers), [TDF: social/professional role and identity].

 “*One day when she lost the baby she was quite down she was on the floor almost… The prescriber rang me she told me look they lost the baby… I saw her she was red orange like she was about to explode I said: “I need to have a chat with you”,*

*[woman receiving OST who lost the child replying] “now I'm not in the mood”,*

*[CP replying]: “yes you are”.*

*and I brought her here in the consultation room and we had a chat when she left I felt she was different. He came as well [the partner] both of them were here I had 20* min *chat with them I had plenty of customers here waiting for me but I said no I'm doing my job properly”* CP.8

### 6.2 Respect

Many CPs regarded their patients as polite and with reasonable conduct and in most cases like any other patient populations. CPs also mentioned the importance of treating patients with respect, to preserve their confidentiality and dignity [TDF: Social/professional role and identity].

*”I accord them respect, I greet them by their name I serve them as promptly as I can umm and some of them have been with me for a number of years so they don't come to me because I am the nearest the most convenient in fact further from the truth they come to me because they are accorded good friendly service“*CP. 15.

### 6.3 Power

Other CPs described having to exhibit authority over this patient population. Partly, because of the legalities of the SC service where pharmacists had to ensure the patient is taking the OST dose without diverting it [TDF: Social / professional role and identity]. Another part was to control cases of drunkenness, aggression and shoplifting [TDF: Social influences]. In one case, one participant said that they had used extended waiting time as a way of punishment for behaviour they deemed unacceptable (e.g. pretending to take an OST dose). Some pharmacists also felt hurt and offended when a patient receiving OST lied or were caught stealing from the pharmacy [TDF: Emotion].

*“You need to be very firm, if they are drunk you say okay go and have a walk and come back so they feel that you are protecting them, even though they might not like it at that moment but they will come back to you and say oh I am sorry about that yesterday and so they like that bit of authority when you can be a bit, kind of umm, so that they know what you are doing is for their own benefit”* CP. 2.

*“You kind of feel a bit hurt* [with reference to shop lifting]*”* CP. 9.

*“Because she lied to me and I don't like to be lied, and I kept her there to remember me…*” CP. 7.

## 7 Theme 4: social influences

*(COM-B: Social Opportunity, Automatic Motivation; [TDF: social influences, emotions])*

CPs commented that they probably would not approach the subject of RH when women receiving OST were accompanied e.g. by children or partners [TDF: Social influences]. CPs felt it was hard to know whether partners supported women with their RH needs or not and that the situation should be assessed very carefully. Some CPs felt that providing SC services might affect the business financially. They assumed that other customers (e.g. older patients or those with children) might feel uncomfortable being around some SC patients. Similar attitudes were perceived regarding sexual health services or advertising them in the pharmacy [TDF: social influences].

*“I don't promote it; I don't put a banner outside saying sexual health clinic because again I want to keep it as for image”* CP. 19.

Some CPs stated that they felt pressure from the pharmacy support staff not to take on more work or felt that pharmacy staff might question the ethics of the service (stigma).CPs described these situations as leaving them struggling to delegate and worried to leave the shop floor for long [TDF: Emotion].

*“CP: I think for the family planning side of things I think they* [the staff] *might struggle a little bit with that but with the contraception thing no problem at all. I think just they would worry they worry about children being brought up in that type of environment.*

*Researcher: How does that make you feel?*

*CP: I understand their concerns I do and sometimes you have them yourself but then you kind of see how others [women receiving OST] cope with, it's almost a split personality in a way that they've got their family life and then they've got this other life [addiction] yeah so and then others you got it's their family nearby who end up looking after a lot so you do see that as well”* CP. 9.

## 8 Theme 5: environmental factors

*(COM-B: Physical Opportunity, Reflective Motivation, Automatic Motivation; [TDF domain: environmental context and resources, beliefs about consequences, social/professional role and identity, reinforcement])*

### 8.1 Workload

Many CPs stated there was a need to have a second pharmacist to cover other services while a service was taking place in the consultation room [TDF: Environmental context and resources]. CPs highlighted the risk of adding many services without freeing the pharmacist from other responsibilities, as this could compromise safety [TDF: Beliefs about consequences]. Additional issues included the burden of paperwork, accessing the summary care record of the patient and having many women receiving OST. In the latter instance, CPs with larger numbers of OST clients felt they would struggle to talk to every female OST patient opportunistically [TDF: Environmental context and resources].

*“We have two pharmacists here most the time four days a week so often we are busy but we still manage to carry out you know the services and stuff”* CP. 3.

### 8.2 Privacy

Most CPs emphasised that the provision of RH service should take place in the consultation room to maintain privacy. However, in some pharmacies the consultation room was very small, far from the dispensary, used for storage or discussion could be heard from outside [TDF: Environmental context and resources]. In other pharmacies, OST patients had to access the pharmacy from a different door with no access to the consultation room. Others used a booth that was not considered sufficiently private. Additionally, some pharmacists were concerned about being in an enclosed area with someone from this patient group because of past negative experiences [TDF: Beliefs about consequences].

*“Most of them have opted to stay out there and just drink it or have it in front of other people. There have been one or two patients who prefer more privacy, so yes, they will come into the room and* [I] *supervise them... they were given a choice”* CP. 10.

### 8.3 Funding

CPs stated that the funding of a RH service would need to depend on how extensive the service would be (e.g. brief advice versus clinical provision) and that it would vary according to local areas' needs. If the service consisted of basic RH advice and referrals to other providers for contraception, pregnancy follow-up or terminations, that was seen as part of the pharmacist's existing role [TDF: Social/professional role and identity]. However, if the service involved more tasks (e.g. prescribing oral contraception, administrating other methods such as depot injections), then it had to be funded as such [TDF: Reinforcement].

*“In terms of the specialist thing that the pharmacist provide in general advice is free basically you don't have to pay for it provided the service but if it's doing anything an extra then I think in general think there might need funding for it but all we can do is signpost to services available for them”* CP. 13.

## 9 Discussion

The main aim of this paper was to provide insights into pharmacists' views on the provision of a RH service to women receiving OST. We used the TDF/COM-B (Fig. 1) to code the influences CPs described on the proposed behaviour.^18^^,^^20^^,^^21^ Nine of the 14 TDF domains (knowledge; memory attention and decision making; social /professional role and identity; beliefs about capabilities; beliefs about consequences; emotion; reinforcement, social influences; environmental context and resources) were identified and mapped to five of the six COM-B elements (Fig. 2).

The analysis approach adopted in this paper allowed for presenting the identified TDF domains and the COM-B elements within a naturally occurring inductive context and provided a better understanding to how these factors affected each other (Fig. 3). For example, CPs' *knowledge* about the topic represented part of their *psychological capability* and could influence their *beliefs about their capabilities*. Knowledge gaps have been identified in this study (OST effects on fertility, CPs' knowledge of the RH needs of women receiving OST). An important observation was that most of CPs were more comfortable if women receiving OST initiated the subject (*social opportunity*) to which they would respond (automatic motivation). When CPs realised that they had to initiate the conversation proactively, CPs' thought processes shifted to their *reflective motivation* (*beliefs about their capabilities and beliefs about consequences*). Another finding was that many CPs emphasised the importance of determining the need for the service for each individual *(psychological capability* [Memory, attention and *decision-making]*).

According to the Andersen healthcare utilization model,^35^ ‘need’ is one of the main factors which lead to use of a healthcare service. In our study, pharmacists talked about selectively offering the intervention, deciding the need ‘on behalf of’ the patient. In the absence of a guideline, our data suggest that some pharmacists may decide on such need (and what advice to give) based on patients' characteristics such as age, marital status, known sex work) and perceived social structure (e.g. if the woman is perceived as capable of taking care of a child). This demonstrates there is a risk that pharmacists would rely on stereotypical judgements about women to determine their need for RH advice, and subsequently lead to inequitable access and stigma.

The findings also pointed to a power dynamic between the pharmacist and all OST patients (not just female patients) and this influenced their interactions. Power dynamics have been studied within a context of physician-patient interaction.^36^^,^^37^ In the physicians' case, their power was a result of them having a *“cultural capital” (*e.g. *medical knowledge & skills) or “symbolic capital” (*e.g. *accumulated professional prestige or honour)”*. However, in the pharmacists' case, in addition to them having a cultural and symbolic capital, the nature of the OST service gave an additional power to the pharmacist which they identified (e.g. the pharmacist's ability to decide whether the patient can take the dose, where they should take it and to refuse to dispense if patient does not comply). With the current emphasis on patient-centeredness and what it means in terms of shared decision-making and dialogue in a health care setting,^35^ the concept of patient-centeredness seems out of reach in this context. In contrast, when other CPs described the relationship with women as good, elements of empathy, trust and respect were apparent in the approach they described for dealing with this patient group. Empathy, trust, respect, and privacy are core pillars of good consultation, and any future service needs to include these pillars in their approach to consulting with women.^38^^,^^39^

## 10 Limitations

One limitation of this study was that the data were collected between Sept 2016 and May 2017. However, the situation in the UK regarding meeting the RH needs of OST female patients remains unchanged. Our findings cannot automatically be generalised to other countries and additional research on the views and experiences of women in other settings would be useful. The CPs responded to an invitation to participate in the study, and therefore may have been more motivated towards the idea of providing RH support for women. Despite this, our data suggests several pharmacists were not motivated to provide this care and thus presenting a variety of opinions.

## 11 Conclusion

This study has identified a range of factors that could influence the provision of reproductive Health (RH) advice/service to women receiving Opioid Substitution Treatment. The study revealed the challenges pharmacists perceived if required to proactively provide a reproductive Health (RH) service, and their perception was subject to reflective motivation (their beliefs about capabilities and consequences). The power dynamics between the pharmacist and the women might act as a barrier to the delivery of the service. The findings, although identified in the context of CPs' suggested provision of RH service to women receiving OST, can be extended to other health care professionals in similar settings, i.e. providing a health care advice can be subjected to the healthcare professional's reflective thinking. Any future RH service design needs to consider the factors identified in this study.

## Funding

This study was funded by a Medical Research Council Public Health Intervention Development (PHIND) award: grant number MR/N011147/1.

## Disclosures/conflict of interest

In the last three years, J.N. has received, through her university, research funding from Mundipharma Research Ltd. and Camurus AB (for unrelated research) and an honorarium from Indivior (for an unrelated conference presentation). J.S. holds a clinical post with Turning Point which supported recruitment for this study, but she does not work in any of the services involved in the research. N.A., A.C. and H.F. have no interests to declare.

## Appendix A Supplementary data

Interview topic guide Image 1
