# Supplementary material for: Community pharmacists' views on providing a reproductive health service to women receiving opioid substitution treatment: A qualitative study using the TDF and COM-B
Source: Explor Res Clin Soc Pharm. 2021 Sep 21;4:100071. doi: 10.1016/j.rcsop.2021.100071 (PMC8626316; doi:10.1016/j.rcsop.2021.100071)
Supplement: Supplementary file 1 — Interview topic guide [file mmc1.docx]

**Community pharmacist provision of contraception services for women receiving opiate substitution treatment**

**Introduction**

- **Thank you** for taking the time to speak to me for this interview.
- It will take **up to 1 hour** to complete, but you can stop at any time you want.
- **Introduce self**, non-judgmental position.
- We would like to hear your **thoughts** and opinions about providing family planning and contraception advice to women receiving opiate substitution. I will ask you about your **relationship** with this group of patients and what you think a contraception **service** provided by pharmacists could **look like**.
- There are **no right or wrong answers**. If you do not want to answer a question, you do not have to, just let me know and we can move on. You can stop the interview at any time as well, but I will ask your permission to keep that data you gave under consent.

**Confidentiality**

Anything you say will be kept completely confidential. If we do use any of your words when we write up the study, we will make sure you cannot be identified.

However, confidentiality will have to be broken if you disclose that you intend to harm yourself or others and that a relevant person will have to be informed

**Housekeeping points**

- I am going to **record** the interview because it is too difficult for me to listen and write everything down at the same time. This is standard when we conduct interviews.
- Do you **have any questions** before we start?
- Are you happy to sign the **consent form** and continue?

Dear Participant

Please answer this short form. They are general questions about yourself so I understand a little bit about you and your circumstances. Please note that you are free to refuse to answer these questions, and that will not affect your participation in this study.

1. **Age:** __________
2. **Gender**: __________
3. **How would you describe your ethnicity?**

| **A. WHITE** | **B. MIXED** |
| --- | --- |
| - British | - White and Black African |
| - Irish | - White and Black Caribbean |
| - Any other white background | - White and Asian |
|  | - Any other Mixed background |
| **C. ASIAN OR ASIAN BRITISH** | **D. BLACK OR BLACK BRITISH** |
| - Bangladeshi | - African |
| - Indian | - Caribbean |
| - Pakistani | - Any other Black background |
| - Any other Asian background |  |
| **E. CHINESE OR OTHER ETHNIC GROUP** | |
| - Chinese - Any other background | |
| ***If any* other *background*** *from sections A-E, please state:*_____________________________________________ | |

**To start the interview, first, I’d like to ask you a few questions about yourself and your role as a community pharmacist**

- 1. How long have you been practising as a community pharmacist?
  2. What is your favourite service? Why is that?

**Next, I have a few questions about the Supervised Administration (SA) service your pharmacy provides**

- 1. How long have you been providing supervised administration?
     1. In your pharmacy?
     2. And as a pharmacist?
  2. How does this service usually run in your pharmacy?
     1. Where in your pharmacy do you provide it?
     2. Who runs it?
  3. What do you think of this service?
     1. What do you think are the benefits of this service?
     2. What parts are challenging?
  4. How many female opiate substitution clients do you have at the moment?
     1. And before?
  5. How would you describe your relationship with your female opiate substitution patients?
     1. Describe to me a patient you feel you established a very good relationship with them? What was that like?
     2. What about a patient where things didn’t go so well? What happened?
  6. How many of your female opiate substitution patients have become pregnant during their time in the SA service?
     1. How often was this planned?
     2. What was the outcome? [birth/ termination/ miscarriage]

**Next, I am interested in finding out what contraception and pregnancy planning advice you think women receiving opiate substitution treatment should receive from their healthcare professionals (Knowledge check)**

- 1. From your perspective, what are the contraception or pregnancy planning needs of your female opiate substitution patients?
  2. What impact do you think opiate substitution treatment has on a woman’s ability to get pregnant?
  3. What do you think the women themselves believe about their ability to get pregnant?
  4. How much control do you feel your female opiate substitution patients have over their contraception?
  5. Describe the support you think the pharmacy can provide for women receiving opiate substitution treatment who want to find out more about contraception or pregnancy planning?
  6. What additional or different advice you would give women receiving opiate substitution treatment on these topics?
  7. What do you believe will happen if you provide contraception or pregnancy planning advice to women receiving opiate substitution treatment?
     1. What do you believe will happen if you don’t provide this advice?
  8. Which forms of contraception would you recommend for women receiving opiate substitution treatment? What makes you say this?
     1. Which forms of contraception wouldn’t you recommend? What makes you say this?

**That’s been very useful so far. I would like now to discuss how to communicate this advice. So:**

- 1. How confident do you feel about providing **contraception** and **pregnancy planning** advice to women receiving opiate substitution treatment?
     1. How confident do you feel broaching this topic?

**If not confident:** what would make you feel more confident?

- - 1. How would you introduce the topic of contraception? What makes you take that approach?
  1. What is your role in providing **contraception or pregnancy planning** advice for women receiving opiate substitution treatment?
     1. What is the role of other pharmacy staff?
     2. What other members of your team do you think would be interested in taking on such a role? Who? Why?
     3. What is the role of other healthcare professionals?
  2. How much do you **want** to provide this advice for women receiving opiate substitution treatment?
  3. What **factors** would make you feel **reluctant** to bring up the topics of contraception and pregnancy planning with women receiving opiate substitution treatment?

Prompts:

- 1. Concerns over complex sexual histories, sex work or abuse?
  2. Mental health issues?
  3. Are there particular types of this patient group for whom providing this advice is more difficult?
  4. Are the particular types of patients from this group who you feel wouldn’t benefit from this advice?
  5. What **factors** do you feel **get in the way** of you providing this advice?

Prompts:

1. how do you balance this role alongside other work in your pharmacy? (competing needs/priorities)
2. how do you balance this against the other needs of this client group?
3. Are there any times of day in your pharmacy when you don’t think it would be possible to provide this advice? (e.g. busy times in your pharmacy)

- 1. What would **help** you to provide this advice?
  2. How do you think women receiving opiate substitution treatment feel/ would feel **about contraception and pregnancy planning advice** from you compared to their other healthcare professionals?
  3. How likely do you think your female OST patients are to ask you for **advice on these topics**?

**My next few questions are focussed on the opportunities for including pregnancy planning and contraception advice into your supervised administration service**

- 1. How would you feel about adding this service to your role or during supervised administration?
  2. What format does / would this support take? (Prompt: e.g. consultation/leaflet/one-to-one consultation, poster etc)
  3. **What** would you like to add to this support if you could in the future?
     1. What are your views on pharmacists providing contraception like the depot injection or the implant in their pharmacy?
  4. **Where** would you provide this advice & support to WRO?
  5. **When** would you give this advice? Why?

(prompt: E.g. at their first visit to your pharmacy for SA or another time?

- 1. How would you feel about **repeating** this advice at a later date with the same patient?
  2. How should a contraception and pregnancy planning service for WRO be **funded?**
     1. For example, would it be by Non-Medical prescribing or Patient Group Direction?
  3. How do you think other community pharmacists will feel about delivering/adding this to their service?
  4. How **practical or feasible** is it for your pharmacy to add this to your service? (prompt; work load, time, paperwork, facilities)
  5. What **other factors** should be considered if planning to introduce such a service more widely?

**My next couple of questions are about your training needs**

- 1. What **training** have you had in providing contraception and pregnancy planning advice to women?
  2. Is there **any other training** or **materials** you **need** to provide this advice?
     1. What do you think the training needs of **other pharmacists** are?
     2. What do you think the training needs of your **pharmacy support staff** are?

**Finally, I have two scenarios and I would like your comments on them**

- 1. Rachel is 28 years old. She has a drink problem and she has been collecting her OST from a city centre pharmacy for the last 6 months. Paul is the pharmacist. He is 58 years old. Paul overheard Rachel telling one of the counter staff that she had an abortion two months ago. Rachel came to the pharmacy twice over the last two months and requested an EHC. The pharmacy provides free EHC under local PGD.

How would you respond? What is your opinion around Rachel’s circumstances?

- 1. A charitable organisation is offering money to women using drugs who agree to either undergo surgical sterilization or use long-acting forms of contraception like Long Acting Reversible Contraception (LARC).

What are your views on this?

**Now we have come to the end of the interview.**

All the information that you have given me will be very useful indeed. Before we end the interview:

8.1. Would you like to add anything else, that we may not have thought to ask about?

8.2 Would you like to ask me any questions?

**Thanks:**

Thank you very much for answering my questions, Thank you for your participation in our study! Your participation is greatly appreciated and valued.

**Debrief**

Go through debrief sheet

**Voucher, locum and travel expenses**

Fill in travel expense form

Fill in locum expense form
